# 3′,6′-Bis(ethyl­amino)-2′,7′-dimethyl-2-{2-(*E*)-[(thio­phen-2-yl)methyl­idene­amino]­eth­yl}spiro­[isoindoline-1,9′-xanthen]-3-one methanol monosolvate

**DOI:** 10.1107/S1600536812018181

**Published:** 2012-04-28

**Authors:** Qing-yu Li, Rui Guo, Zhi-hong Xu

**Affiliations:** a College of Urban Planning and Environmental Science, Xuchang University, Xuchang, Henan Province 461000, People’s Republic of China; b School of Chemistry and Chemical Engineering, Xuchang University, Xuchang, Henan Province 461000, People’s Republic of China

## Abstract

The title compound, C_33_H_34_N_4_O_2_S·CH_3_OH, was prepared as a spiro­lactam ring formation of rhodamine 6 G dye for comparison with a ring-opened form. The xanthene and spiro­lactam rings are approximately planar [r.m.s. deviations from planarity = 0.122 (3) and 0.072 (6) Å, respectively]. The dihedral angles formed by the spiro­lactam and thio­phene rings with the xanthene ring system are 89.7 (6) and 86.5 (2)°, respectively. The crystal structure features N—H⋯O and C—H⋯O hydrogen bonds.

## Related literature

For rhodamine derivatives bearing a lactam moiety, see: Tian & Peng (2008 ▶); Wu *et al.* (2007 ▶); Xi *et al.* (2011 ▶); Xu *et al.* (2009 ▶, 2011 ▶); Xu, Guo *et al.* (2010 ▶); Xu, Zhang *et al.* (2010 ▶); Zhang *et al.* (2008 ▶).
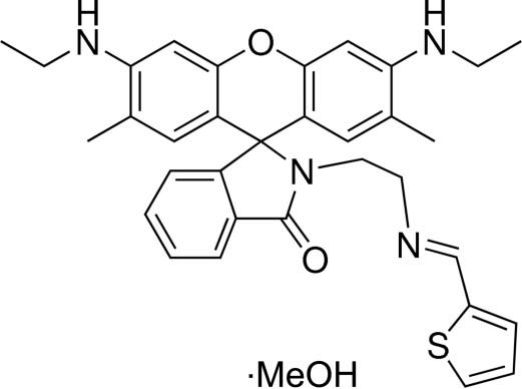


## Experimental

### 

#### Crystal data

C_33_H_34_N_4_O_2_S·CH_4_O
*M* *_r_* = 582.74
Monoclinic, 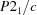

*a* = 9.287 (2) Å
*b* = 9.493 (2) Å
*c* = 35.754 (8) Å
β = 95.683 (4)°
*V* = 3136.8 (12) Å^3^
*Z* = 4
Mo *K*α radiation
μ = 0.14 mm^−1^
*T* = 296 K
0.25 × 0.23 × 0.18 mm

#### Data collection

Bruker SMART CCD area-detector diffractometer
Absorption correction: multi-scan (*SADABS*; Bruker, 2005 ▶) *T*_min_ = 0.965, *T*_max_ = 0.975
15561 measured reflections
5610 independent reflections
3564 reflections with *I* > 2σ(*I*)
*R*_int_ = 0.035

#### Refinement

*R*[*F* ^2^ > 2σ(*F* ^2^)] = 0.077
*wR*(*F* ^2^) = 0.250
*S* = 1.03
5610 reflections
385 parameters
35 restraints
H-atom parameters constrained
Δρ_max_ = 1.10 e Å^−3^
Δρ_min_ = −0.56 e Å^−3^

### 

Data collection: *SMART* (Bruker, 2005 ▶); cell refinement: *SAINT* (Bruker, 2005 ▶); data reduction: *SAINT*; program(s) used to solve structure: *SHELXS97* (Sheldrick, 2008 ▶); program(s) used to refine structure: *SHELXL97* (Sheldrick, 2008 ▶); molecular graphics: *SHELXTL* (Sheldrick, 2008 ▶); software used to prepare material for publication: *SHELXTL*.

## Supplementary Material

Crystal structure: contains datablock(s) I, global. DOI: 10.1107/S1600536812018181/zj2071sup1.cif

Structure factors: contains datablock(s) I. DOI: 10.1107/S1600536812018181/zj2071Isup2.hkl

Additional supplementary materials:  crystallographic information; 3D view; checkCIF report

## Figures and Tables

**Table 1:** Hydrogen-bond geometry (Å, °)

*D*—H⋯*A*	*D*—H	H⋯*A*	*D*⋯*A*	*D*—H⋯*A*
N2—H2⋯O3^i^	0.86	2.27	3.108	166
C5—H5⋯O2^ii^	0.93	2.44	3.360	170

Symmetry codes: (i) 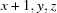; (ii) 
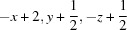
.

**Fig. 1.**
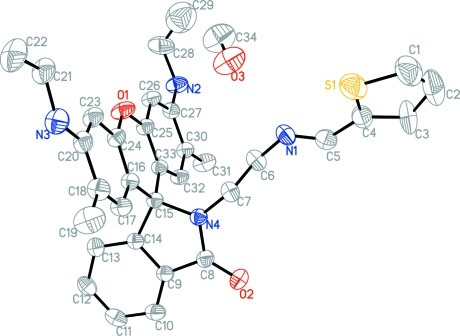
The molecular structure of the title compound, with displacement ellipsoids drawn at 30% probability level.

**Fig. 2.**
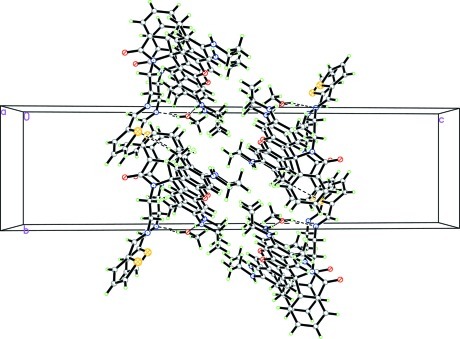
The packing diagram of the compound and the solvent methanol molecular view down a-axis.

## supplementary crystallographic information

### Comment

Among many fluorescent compounds, rhodamine dyes are known to have excellent photophysical properties, and they are one of the most widely used fluorophores for labeling and sensing biomolecules. There are a few single-crystal reports about rhodamine derivatives bearing a lactam moiety (Xu *et al.*, 2009; Xi *et al.*, 2011; Xu *et al.*, 2011; Wu *et al.*, 2007; Xu, Zhang *et al.*, 2010; Xu, Guo *et al.*, 2010; Zhang *et al.*, 2008; Tian *et al.*, 2008;). Detailed information on their molecular and crystal structures is necessary to understand their photophysical and photochemical properties.

In agreement with other reported models, (Xu *et al.*, 2009; Wu *et al.*, 2007; Zhang *et al.*, 2008; Tian *et al.*, 2008; Xi *et al.*, 2011;) the main skeleton of the molecule is formed by the xanthene ring and the spirolactam-ring. As shown in Figure 1, The atoms of the xanthene ring or the spirolactam-ring are both nearly planar and are almost perpendicular to each other. The dihedral angle between the xanthene mean planes and the spirolactam ring fragment is 89.7 (6)°. The dihedral angle between the xanthene mean planes and the thiophene ring is 86.5 (2)°.

### Experimental

A portion of *N*-(rhodamine-6 G)lactam-ethylenediamine (228 mg, 0.5 mmol) and thiophenecarboxaldehyde (57.7 mg, 0.6 mmol) were combined in fresh distilled acetonitrile (50 ml). The reaction solution was refluxed for 24 h under N2 atmosphere. After that, the solution was cooled (concentrated to 10 ml) and allowed to stand at room temperature overnight. The precipitate which appeared next day was filtered and the crude product was purified by recrystallization from acetonitrile to give 247.6 mg of the title compound in 90% yield. Single crystals suitable for X-ray measurements were obtained from reaction mother liquid by slow evaporation at room temperature.

### Refinement

The H atoms attached to C, N and O atoms were placed in geometrically calculated positions (C—H = 0.93–0.97 Å and O—H = 0.82 Å) and refined as riding, with *U*_iso_(H) = 1.2*U*_eq_(C, N) or 1.5*U*_eq_(methyl C, O).

### Crystal data

**Table:**

C_33_H_34_N_4_O_2_S·CH_4_O	*F*(000) = 1240
*M**_r_* = 582.74	*D*_x_ = 1.234 Mg m^−^^3^
Monoclinic, *P*2_1_/*c*	Mo *K*α radiation, λ = 0.71073 Å
*a* = 9.287 (2) Å	Cell parameters from 3316 reflections
*b* = 9.493 (2) Å	µ = 0.14 mm^−^^1^
*c* = 35.754 (8) Å	*T* = 296 K
β = 95.683 (4)°	Block, colorless
*V* = 3136.8 (12) Å^3^	0.25 × 0.23 × 0.18 mm
*Z* = 4	

### Data collection

**Table:**

Bruker SMART CCD area-detector diffractometer	5610 independent reflections
Radiation source: fine-focus sealed tube	3564 reflections with *I* > 2σ(*I*)
Graphite monochromator	*R*_int_ = 0.035
phi and ω scans	θ_max_ = 25.2°, θ_min_ = 2.2°
Absorption correction: multi-scan (*SADABS*; Bruker, 2005)	*h* = −11→11
*T*_min_ = 0.965, *T*_max_ = 0.975	*k* = −11→11
15561 measured reflections	*l* = −42→24

### Refinement

**Table:**

Refinement on *F*^2^	Primary atom site location: structure-invariant direct methods
Least-squares matrix: full	Secondary atom site location: difference Fourier map
*R*[*F*^2^ > 2σ(*F*^2^)] = 0.077	Hydrogen site location: inferred from neighbouring sites
*wR*(*F*^2^) = 0.250	H-atom parameters constrained
*S* = 1.03	*w* = 1/[σ^2^(*F*_o_^2^) + (0.1391*P*)^2^ + 1.4198*P*] where *P* = (*F*_o_^2^ + 2*F*_c_^2^)/3
5610 reflections	(Δ/σ)_max_ < 0.001
385 parameters	Δρ_max_ = 1.10 e Å^−^^3^
35 restraints	Δρ_min_ = −0.56 e Å^−^^3^

### Special details

**Table:**

Geometry. All e.s.d.'s (except the e.s.d. in the dihedral angle between two l.s. planes) are estimated using the full covariance matrix. The cell e.s.d.'s are taken into account individually in the estimation of e.s.d.'s in distances, angles and torsion angles; correlations between e.s.d.'s in cell parameters are only used when they are defined by crystal symmetry. An approximate (isotropic) treatment of cell e.s.d.'s is used for estimating e.s.d.'s involving l.s. planes.
Refinement. Refinement of *F*^2^ against ALL reflections. The weighted *R*-factor *wR* and goodness of fit *S* are based on *F*^2^, conventional *R*-factors *R* are based on *F*, with *F* set to zero for negative *F*^2^. The threshold expression of *F*^2^ > σ(*F*^2^) is used only for calculating *R*-factors(gt) *etc*. and is not relevant to the choice of reflections for refinement. *R*-factors based on *F*^2^ are statistically about twice as large as those based on *F*, and *R*- factors based on ALL data will be even larger.

### Fractional atomic coordinates and isotropic or equivalent isotropic displacement parameters (Å^2^)

**Table:**

	*x*	*y*	*z*	*U*_iso_*/*U*_eq_	
N3	0.3532 (3)	0.0575 (4)	0.04285 (10)	0.0731 (9)	
H3	0.2884	−0.0001	0.0492	0.088*	
C21	0.3228 (4)	0.1405 (5)	0.01026 (12)	0.0769 (12)	
H21A	0.3916	0.1190	−0.0076	0.092*	
H21B	0.3326	0.2395	0.0168	0.092*	
C22	0.1706 (5)	0.1118 (6)	−0.00738 (14)	0.1012 (17)	
H22A	0.1629	0.0151	−0.0152	0.152*	
H22B	0.1495	0.1720	−0.0288	0.152*	
H22C	0.1030	0.1300	0.0107	0.152*	
N2	1.2815 (3)	0.4693 (3)	0.07386 (9)	0.0700 (9)	
H2	1.3620	0.4874	0.0870	0.084*	
C28	1.2508 (6)	0.5386 (6)	0.03806 (16)	0.0947 (15)	
H28A	1.3418	0.5698	0.0297	0.114*	
H28B	1.2103	0.4692	0.0201	0.114*	
C29	1.1596 (11)	0.6506 (11)	0.0368 (3)	0.181 (3)	
H29A	1.0626	0.6183	0.0386	0.272*	
H29B	1.1637	0.6999	0.0134	0.272*	
H29C	1.1881	0.7130	0.0573	0.272*	
S1	0.50637 (16)	0.71838 (15)	0.19815 (4)	0.1033 (5)	
O1	0.8277 (3)	0.2390 (3)	0.06317 (7)	0.0677 (8)	
C16	0.7508 (3)	0.0775 (3)	0.10973 (9)	0.0468 (8)	
C33	0.9946 (3)	0.1860 (3)	0.11752 (8)	0.0448 (7)	
C14	0.9616 (3)	−0.0529 (3)	0.14470 (9)	0.0437 (7)	
C25	0.9593 (3)	0.2555 (3)	0.08435 (9)	0.0493 (8)	
C30	1.2295 (3)	0.3048 (4)	0.12193 (9)	0.0508 (8)	
N4	0.8638 (3)	0.1385 (3)	0.17287 (7)	0.0463 (6)	
C20	0.4841 (4)	0.0657 (4)	0.06497 (10)	0.0577 (9)	
C27	1.1875 (3)	0.3756 (3)	0.08784 (9)	0.0516 (8)	
C17	0.6351 (4)	−0.0026 (4)	0.11972 (10)	0.0573 (9)	
H17	0.6481	−0.0545	0.1419	0.069*	
C9	0.9808 (3)	−0.0692 (3)	0.18313 (9)	0.0477 (8)	
O2	0.9118 (3)	0.0733 (3)	0.23449 (7)	0.0712 (7)	
C23	0.5967 (4)	0.1470 (4)	0.05452 (10)	0.0589 (9)	
H23	0.5848	0.1982	0.0322	0.071*	
C8	0.9173 (3)	0.0521 (4)	0.20091 (9)	0.0511 (8)	
C24	0.7267 (3)	0.1532 (4)	0.07680 (9)	0.0515 (8)	
C26	1.0530 (4)	0.3494 (4)	0.06969 (10)	0.0579 (9)	
H26	1.0243	0.3954	0.0472	0.070*	
C15	0.8912 (3)	0.0887 (3)	0.13460 (8)	0.0436 (7)	
C32	1.1319 (3)	0.2136 (3)	0.13547 (9)	0.0502 (8)	
H32	1.1592	0.1675	0.1580	0.060*	
C12	1.0738 (4)	−0.2726 (4)	0.13768 (12)	0.0616 (10)	
H12	1.1052	−0.3431	0.1224	0.074*	
C13	1.0076 (3)	−0.1542 (4)	0.12131 (10)	0.0540 (8)	
H13	0.9947	−0.1437	0.0953	0.065*	
C7	0.7759 (4)	0.2598 (4)	0.17917 (11)	0.0559 (9)	
H7A	0.7385	0.2495	0.2034	0.067*	
H7B	0.6938	0.2609	0.1602	0.067*	
N1	0.7468 (3)	0.5095 (3)	0.18310 (9)	0.0635 (8)	
C11	1.0941 (4)	−0.2885 (4)	0.17591 (13)	0.0669 (10)	
H11	1.1398	−0.3689	0.1862	0.080*	
C5	0.7711 (4)	0.5849 (4)	0.21151 (12)	0.0659 (10)	
H5	0.8553	0.5697	0.2273	0.079*	
C10	1.0479 (4)	−0.1873 (4)	0.19906 (11)	0.0621 (10)	
H10	1.0615	−0.1978	0.2250	0.074*	
C3	0.7036 (6)	0.7954 (6)	0.25073 (17)	0.1065 (18)	
H3A	0.7857	0.7988	0.2679	0.128*	
C4	0.6718 (5)	0.6968 (4)	0.22100 (12)	0.0725 (11)	
C6	0.8522 (4)	0.3990 (4)	0.17837 (12)	0.0641 (10)	
H6A	0.9303	0.4035	0.1985	0.077*	
H6B	0.8930	0.4108	0.1546	0.077*	
C1	0.4698 (8)	0.8549 (7)	0.2229 (2)	0.121 (2)	
H1	0.3823	0.9031	0.2198	0.145*	
C2	0.5783 (10)	0.8903 (7)	0.2483 (2)	0.142 (3)	
H2A	0.5750	0.9697	0.2634	0.170*	
O3	0.5909 (4)	0.5509 (5)	0.10809 (10)	0.1090 (12)	
H3B	0.6220	0.5510	0.1304	0.164*	
C34	0.6938 (7)	0.6016 (7)	0.08725 (18)	0.126 (2)	
H34A	0.6659	0.6935	0.0779	0.188*	
H34B	0.7038	0.5394	0.0665	0.188*	
H34C	0.7844	0.6078	0.1026	0.188*	
C18	0.5032 (4)	−0.0097 (4)	0.09893 (11)	0.0629 (10)	
C19	0.3811 (5)	−0.0949 (6)	0.11157 (15)	0.0973 (16)	
H19A	0.4152	−0.1482	0.1335	0.146*	
H19B	0.3453	−0.1581	0.0918	0.146*	
H19C	0.3048	−0.0331	0.1175	0.146*	
C31	1.3756 (4)	0.3270 (5)	0.14240 (12)	0.0770 (12)	
H31A	1.3874	0.2660	0.1639	0.116*	
H31B	1.3851	0.4233	0.1505	0.116*	
H31C	1.4485	0.3060	0.1260	0.116*	

### Atomic displacement parameters (Å^2^)

**Table:**

	*U*^11^	*U*^22^	*U*^33^	*U*^12^	*U*^13^	*U*^23^
N3	0.0471 (17)	0.087 (2)	0.080 (2)	−0.0115 (16)	−0.0174 (15)	−0.0034 (19)
C21	0.057 (2)	0.093 (3)	0.075 (3)	0.004 (2)	−0.020 (2)	−0.011 (2)
C22	0.058 (3)	0.143 (5)	0.095 (3)	0.007 (3)	−0.027 (2)	−0.024 (3)
N2	0.0590 (18)	0.079 (2)	0.071 (2)	−0.0209 (16)	0.0008 (15)	0.0240 (17)
C28	0.081 (3)	0.088 (3)	0.114 (4)	−0.021 (3)	0.004 (3)	0.040 (3)
C29	0.193 (9)	0.179 (8)	0.170 (8)	−0.020 (7)	0.005 (7)	0.007 (7)
S1	0.1007 (10)	0.0925 (9)	0.1156 (11)	0.0222 (7)	0.0048 (8)	0.0020 (8)
O1	0.0522 (14)	0.0884 (18)	0.0585 (14)	−0.0218 (13)	−0.0145 (11)	0.0252 (13)
C16	0.0396 (16)	0.0493 (18)	0.0501 (18)	−0.0038 (14)	−0.0019 (13)	0.0029 (15)
C33	0.0427 (17)	0.0447 (17)	0.0462 (17)	−0.0008 (13)	0.0002 (13)	0.0029 (14)
C14	0.0352 (15)	0.0459 (17)	0.0492 (18)	−0.0076 (13)	0.0003 (13)	0.0038 (14)
C25	0.0438 (17)	0.0559 (19)	0.0467 (18)	−0.0054 (14)	−0.0028 (14)	0.0044 (15)
C30	0.0428 (17)	0.0556 (19)	0.0527 (19)	−0.0068 (15)	−0.0022 (14)	0.0047 (15)
N4	0.0449 (14)	0.0462 (15)	0.0472 (14)	−0.0008 (12)	0.0013 (11)	0.0020 (12)
C20	0.0448 (18)	0.063 (2)	0.062 (2)	−0.0004 (16)	−0.0102 (15)	−0.0086 (18)
C27	0.0465 (18)	0.0512 (19)	0.058 (2)	−0.0066 (15)	0.0075 (15)	0.0026 (16)
C17	0.0484 (19)	0.061 (2)	0.061 (2)	−0.0095 (16)	−0.0030 (16)	0.0100 (17)
C9	0.0455 (17)	0.0466 (18)	0.0491 (18)	−0.0084 (14)	−0.0049 (14)	0.0044 (15)
O2	0.0900 (19)	0.0758 (17)	0.0470 (14)	−0.0010 (15)	0.0027 (13)	−0.0039 (13)
C23	0.0492 (19)	0.070 (2)	0.055 (2)	−0.0055 (17)	−0.0098 (16)	0.0029 (17)
C8	0.0501 (18)	0.0543 (19)	0.0473 (19)	−0.0087 (15)	−0.0027 (14)	0.0006 (16)
C24	0.0426 (17)	0.061 (2)	0.0496 (18)	−0.0058 (15)	−0.0009 (14)	−0.0006 (16)
C26	0.058 (2)	0.063 (2)	0.0512 (19)	−0.0083 (17)	−0.0012 (16)	0.0146 (17)
C15	0.0397 (16)	0.0460 (17)	0.0442 (16)	−0.0030 (13)	−0.0010 (13)	0.0025 (14)
C32	0.0427 (17)	0.056 (2)	0.0505 (18)	−0.0038 (15)	−0.0026 (14)	0.0101 (15)
C12	0.050 (2)	0.050 (2)	0.085 (3)	0.0005 (16)	0.0104 (19)	−0.0049 (19)
C13	0.0457 (18)	0.056 (2)	0.060 (2)	−0.0050 (15)	0.0064 (15)	−0.0048 (17)
C7	0.0488 (19)	0.053 (2)	0.065 (2)	0.0001 (15)	0.0029 (16)	−0.0059 (16)
N1	0.0647 (19)	0.0474 (16)	0.077 (2)	0.0011 (14)	−0.0005 (16)	−0.0065 (16)
C11	0.057 (2)	0.050 (2)	0.093 (3)	0.0025 (17)	0.004 (2)	0.014 (2)
C5	0.060 (2)	0.063 (2)	0.075 (3)	−0.0038 (18)	0.0062 (19)	0.005 (2)
C10	0.061 (2)	0.058 (2)	0.065 (2)	−0.0041 (18)	−0.0041 (18)	0.0150 (19)
C3	0.093 (3)	0.103 (4)	0.125 (4)	−0.001 (3)	0.018 (3)	−0.066 (3)
C4	0.080 (3)	0.063 (2)	0.077 (3)	−0.003 (2)	0.021 (2)	−0.004 (2)
C6	0.057 (2)	0.053 (2)	0.082 (3)	−0.0007 (17)	0.0056 (18)	−0.0047 (19)
C1	0.120 (5)	0.108 (5)	0.140 (5)	0.032 (4)	0.040 (4)	−0.004 (4)
C2	0.159 (7)	0.115 (5)	0.159 (6)	0.019 (5)	0.053 (5)	−0.055 (5)
O3	0.078 (2)	0.149 (3)	0.097 (2)	−0.047 (2)	−0.0066 (18)	0.023 (2)
C34	0.099 (4)	0.158 (6)	0.121 (5)	−0.034 (4)	0.015 (3)	0.023 (4)
C18	0.0433 (19)	0.067 (2)	0.077 (3)	−0.0107 (17)	−0.0006 (17)	0.003 (2)
C19	0.059 (3)	0.127 (4)	0.103 (3)	−0.034 (3)	−0.007 (2)	0.022 (3)
C31	0.054 (2)	0.097 (3)	0.077 (3)	−0.025 (2)	−0.0080 (19)	0.022 (2)

### Geometric parameters (Å, º)

**Table:**

N3—C20	1.385 (4)	C17—H17	0.9300
N3—C21	1.412 (5)	C9—C10	1.379 (5)
N3—H3	0.8600	C9—C8	1.466 (5)
C21—C22	1.515 (5)	O2—C8	1.223 (4)
C21—H21A	0.9700	C23—C24	1.381 (4)
C21—H21B	0.9700	C23—H23	0.9300
C22—H22A	0.9600	C26—H26	0.9300
C22—H22B	0.9600	C32—H32	0.9300
C22—H22C	0.9600	C12—C11	1.370 (6)
N2—C27	1.375 (4)	C12—C13	1.383 (5)
N2—C28	1.442 (5)	C12—H12	0.9300
N2—H2	0.8600	C13—H13	0.9300
C28—C29	1.357 (10)	C7—C6	1.502 (5)
C28—H28A	0.9700	C7—H7A	0.9700
C28—H28B	0.9700	C7—H7B	0.9700
C29—H29A	0.9600	N1—C5	1.245 (5)
C29—H29B	0.9600	N1—C6	1.456 (5)
C29—H29C	0.9600	C11—C10	1.365 (5)
S1—C1	1.625 (7)	C11—H11	0.9300
S1—C4	1.680 (5)	C5—C4	1.468 (6)
O1—C24	1.368 (4)	C5—H5	0.9300
O1—C25	1.381 (4)	C10—H10	0.9300
C16—C24	1.379 (4)	C3—C4	1.425 (6)
C16—C17	1.392 (4)	C3—C2	1.468 (9)
C16—C15	1.507 (4)	C3—H3A	0.9300
C33—C25	1.368 (4)	C6—H6A	0.9700
C33—C32	1.394 (4)	C6—H6B	0.9700
C33—C15	1.505 (4)	C1—C2	1.330 (9)
C14—C13	1.370 (5)	C1—H1	0.9300
C14—C9	1.377 (4)	C2—H2A	0.9300
C14—C15	1.522 (4)	O3—C34	1.357 (6)
C25—C26	1.384 (5)	O3—H3B	0.8200
C30—C32	1.376 (4)	C34—H34A	0.9600
C30—C27	1.413 (5)	C34—H34B	0.9600
C30—C31	1.492 (5)	C34—H34C	0.9600
N4—C8	1.351 (4)	C18—C19	1.498 (5)
N4—C7	1.442 (4)	C19—H19A	0.9600
N4—C15	1.493 (4)	C19—H19B	0.9600
C20—C23	1.381 (5)	C19—H19C	0.9600
C20—C18	1.405 (5)	C31—H31A	0.9600
C27—C26	1.372 (5)	C31—H31B	0.9600
C17—C18	1.370 (5)	C31—H31C	0.9600
			
C20—N3—C21	122.1 (3)	C25—C26—H26	119.5
C20—N3—H3	118.9	N4—C15—C33	110.5 (2)
C21—N3—H3	118.9	N4—C15—C16	110.3 (2)
N3—C21—C22	110.2 (4)	C33—C15—C16	110.7 (2)
N3—C21—H21A	109.6	N4—C15—C14	99.8 (2)
C22—C21—H21A	109.6	C33—C15—C14	111.2 (2)
N3—C21—H21B	109.6	C16—C15—C14	113.8 (2)
C22—C21—H21B	109.6	C30—C32—C33	124.1 (3)
H21A—C21—H21B	108.1	C30—C32—H32	117.9
C21—C22—H22A	109.5	C33—C32—H32	117.9
C21—C22—H22B	109.5	C11—C12—C13	121.6 (4)
H22A—C22—H22B	109.5	C11—C12—H12	119.2
C21—C22—H22C	109.5	C13—C12—H12	119.2
H22A—C22—H22C	109.5	C14—C13—C12	117.7 (3)
H22B—C22—H22C	109.5	C14—C13—H13	121.2
C27—N2—C28	122.7 (3)	C12—C13—H13	121.2
C27—N2—H2	118.6	N4—C7—C6	115.1 (3)
C28—N2—H2	118.6	N4—C7—H7A	108.5
C29—C28—N2	117.1 (6)	C6—C7—H7A	108.5
C29—C28—H28A	108.0	N4—C7—H7B	108.5
N2—C28—H28A	108.0	C6—C7—H7B	108.5
C29—C28—H28B	108.0	H7A—C7—H7B	107.5
N2—C28—H28B	108.0	C5—N1—C6	116.0 (3)
H28A—C28—H28B	107.3	C10—C11—C12	120.4 (3)
C28—C29—H29A	109.5	C10—C11—H11	119.8
C28—C29—H29B	109.5	C12—C11—H11	119.8
H29A—C29—H29B	109.5	N1—C5—C4	122.5 (4)
C28—C29—H29C	109.5	N1—C5—H5	118.7
H29A—C29—H29C	109.5	C4—C5—H5	118.7
H29B—C29—H29C	109.5	C11—C10—C9	118.6 (3)
C1—S1—C4	93.6 (3)	C11—C10—H10	120.7
C24—O1—C25	118.2 (2)	C9—C10—H10	120.7
C24—C16—C17	116.1 (3)	C4—C3—C2	104.7 (5)
C24—C16—C15	121.6 (3)	C4—C3—H3A	127.6
C17—C16—C15	122.1 (3)	C2—C3—H3A	127.6
C25—C33—C32	116.0 (3)	C3—C4—C5	124.0 (4)
C25—C33—C15	122.7 (3)	C3—C4—S1	113.3 (4)
C32—C33—C15	121.3 (3)	C5—C4—S1	122.8 (3)
C13—C14—C9	120.9 (3)	N1—C6—C7	107.9 (3)
C13—C14—C15	128.9 (3)	N1—C6—H6A	110.1
C9—C14—C15	110.3 (3)	C7—C6—H6A	110.1
C33—C25—O1	122.9 (3)	N1—C6—H6B	110.1
C33—C25—C26	122.2 (3)	C7—C6—H6B	110.1
O1—C25—C26	114.9 (3)	H6A—C6—H6B	108.4
C32—C30—C27	117.9 (3)	C2—C1—S1	112.8 (5)
C32—C30—C31	121.0 (3)	C2—C1—H1	123.6
C27—C30—C31	121.1 (3)	S1—C1—H1	123.6
C8—N4—C7	122.8 (3)	C1—C2—C3	115.3 (6)
C8—N4—C15	113.9 (3)	C1—C2—H2A	122.3
C7—N4—C15	123.1 (3)	C3—C2—H2A	122.3
C23—C20—N3	121.5 (3)	C34—O3—H3B	109.5
C23—C20—C18	119.0 (3)	O3—C34—H34A	109.5
N3—C20—C18	119.5 (3)	O3—C34—H34B	109.5
C26—C27—N2	121.8 (3)	H34A—C34—H34B	109.5
C26—C27—C30	118.8 (3)	O3—C34—H34C	109.5
N2—C27—C30	119.4 (3)	H34A—C34—H34C	109.5
C18—C17—C16	124.0 (3)	H34B—C34—H34C	109.5
C18—C17—H17	118.0	C17—C18—C20	118.2 (3)
C16—C17—H17	118.0	C17—C18—C19	121.7 (4)
C14—C9—C10	120.8 (3)	C20—C18—C19	120.1 (3)
C14—C9—C8	109.1 (3)	C18—C19—H19A	109.5
C10—C9—C8	130.1 (3)	C18—C19—H19B	109.5
C20—C23—C24	120.7 (3)	H19A—C19—H19B	109.5
C20—C23—H23	119.7	C18—C19—H19C	109.5
C24—C23—H23	119.7	H19A—C19—H19C	109.5
O2—C8—N4	125.5 (3)	H19B—C19—H19C	109.5
O2—C8—C9	127.7 (3)	C30—C31—H31A	109.5
N4—C8—C9	106.8 (3)	C30—C31—H31B	109.5
O1—C24—C16	123.8 (3)	H31A—C31—H31B	109.5
O1—C24—C23	114.3 (3)	C30—C31—H31C	109.5
C16—C24—C23	122.0 (3)	H31A—C31—H31C	109.5
C27—C26—C25	121.0 (3)	H31B—C31—H31C	109.5
C27—C26—H26	119.5		
			
C20—N3—C21—C22	−178.4 (4)	C25—C33—C15—N4	−120.9 (3)
C27—N2—C28—C29	79.0 (7)	C32—C33—C15—N4	57.2 (4)
C32—C33—C25—O1	179.7 (3)	C25—C33—C15—C16	1.7 (4)
C15—C33—C25—O1	−2.1 (5)	C32—C33—C15—C16	179.8 (3)
C32—C33—C25—C26	−1.0 (5)	C25—C33—C15—C14	129.2 (3)
C15—C33—C25—C26	177.2 (3)	C32—C33—C15—C14	−52.7 (4)
C24—O1—C25—C33	2.5 (5)	C24—C16—C15—N4	120.7 (3)
C24—O1—C25—C26	−176.8 (3)	C17—C16—C15—N4	−55.2 (4)
C21—N3—C20—C23	−5.7 (6)	C24—C16—C15—C33	−2.0 (4)
C21—N3—C20—C18	174.1 (4)	C17—C16—C15—C33	−177.9 (3)
C28—N2—C27—C26	−4.7 (6)	C24—C16—C15—C14	−128.1 (3)
C28—N2—C27—C30	175.7 (4)	C17—C16—C15—C14	56.0 (4)
C32—C30—C27—C26	−0.7 (5)	C13—C14—C15—N4	177.2 (3)
C31—C30—C27—C26	178.6 (4)	C9—C14—C15—N4	−4.9 (3)
C32—C30—C27—N2	178.9 (3)	C13—C14—C15—C33	−66.1 (4)
C31—C30—C27—N2	−1.8 (5)	C9—C14—C15—C33	111.7 (3)
C24—C16—C17—C18	0.3 (5)	C13—C14—C15—C16	59.7 (4)
C15—C16—C17—C18	176.4 (3)	C9—C14—C15—C16	−122.4 (3)
C13—C14—C9—C10	0.6 (5)	C27—C30—C32—C33	0.4 (5)
C15—C14—C9—C10	−177.5 (3)	C31—C30—C32—C33	−178.9 (4)
C13—C14—C9—C8	−178.1 (3)	C25—C33—C32—C30	0.4 (5)
C15—C14—C9—C8	3.8 (3)	C15—C33—C32—C30	−177.9 (3)
N3—C20—C23—C24	−179.6 (3)	C9—C14—C13—C12	−0.1 (4)
C18—C20—C23—C24	0.7 (6)	C15—C14—C13—C12	177.6 (3)
C7—N4—C8—O2	−8.3 (5)	C11—C12—C13—C14	−0.5 (5)
C15—N4—C8—O2	177.7 (3)	C8—N4—C7—C6	103.6 (4)
C7—N4—C8—C9	171.4 (3)	C15—N4—C7—C6	−82.9 (4)
C15—N4—C8—C9	−2.6 (3)	C13—C12—C11—C10	0.6 (5)
C14—C9—C8—O2	178.9 (3)	C6—N1—C5—C4	−177.4 (3)
C10—C9—C8—O2	0.3 (6)	C12—C11—C10—C9	−0.1 (5)
C14—C9—C8—N4	−0.8 (3)	C14—C9—C10—C11	−0.5 (5)
C10—C9—C8—N4	−179.4 (3)	C8—C9—C10—C11	177.9 (3)
C25—O1—C24—C16	−2.9 (5)	C2—C3—C4—C5	177.0 (5)
C25—O1—C24—C23	177.7 (3)	C2—C3—C4—S1	−4.4 (6)
C17—C16—C24—O1	178.9 (3)	N1—C5—C4—C3	−172.5 (5)
C15—C16—C24—O1	2.8 (5)	N1—C5—C4—S1	9.0 (6)
C17—C16—C24—C23	−1.7 (5)	C1—S1—C4—C3	2.2 (4)
C15—C16—C24—C23	−177.8 (3)	C1—S1—C4—C5	−179.1 (4)
C20—C23—C24—O1	−179.3 (3)	C5—N1—C6—C7	118.2 (4)
C20—C23—C24—C16	1.2 (6)	N4—C7—C6—N1	176.9 (3)
N2—C27—C26—C25	−179.5 (3)	C4—S1—C1—C2	1.0 (6)
C30—C27—C26—C25	0.1 (5)	S1—C1—C2—C3	−4.0 (9)
C33—C25—C26—C27	0.8 (6)	C4—C3—C2—C1	5.3 (8)
O1—C25—C26—C27	−179.9 (3)	C16—C17—C18—C20	1.5 (6)
C8—N4—C15—C33	−112.6 (3)	C16—C17—C18—C19	−178.4 (4)
C7—N4—C15—C33	73.4 (3)	C23—C20—C18—C17	−2.0 (5)
C8—N4—C15—C16	124.6 (3)	N3—C20—C18—C17	178.2 (3)
C7—N4—C15—C16	−49.4 (4)	C23—C20—C18—C19	178.0 (4)
C8—N4—C15—C14	4.6 (3)	N3—C20—C18—C19	−1.8 (6)
C7—N4—C15—C14	−169.5 (3)		

### Hydrogen-bond geometry (Å, º)

**Table:**

*D*—H···*A*	*D*—H	H···*A*	*D*···*A*	*D*—H···*A*
N2—H2···O3^i^	0.86	2.27	3.108	166
C5—H5···O2^ii^	0.93	2.44	3.360	170

Symmetry codes: (i) *x*+1, *y*, *z*; (ii) −*x*+2, *y*+1/2, −*z*+1/2.
